# The variation of acute treatment costs of trauma in high-income countries

**DOI:** 10.1186/1472-6963-12-267

**Published:** 2012-08-21

**Authors:** Lynsey Willenberg, Kate Curtis, Colman Taylor, Stephen Jan, Parisa Glass, John Myburgh

**Affiliations:** 1 The George Institute for Global Health, Kent St, Sydney, Australia; 2 Sydney nursing school, University of Sydney, 88 Mallet St, Camperdown, Australia; 3 St George Hospital, Gray St, Kogarah, Australia; 4 Faculty of Medicine, University of New South Wales, Sydney, Australia

**Keywords:** Wounds and injuries, Hospital costs, Economics, Medical, Cost allocations, Cost and cost analysis

## Abstract

**Background:**

In order to assist health service planning, understanding factors that influence higher trauma treatment costs is essential. The majority of trauma costing research reports the cost of trauma from the perspective of the receiving hospital. There has been no comprehensive synthesis and little assessment of the drivers of cost variation, such as country, trauma, subgroups and methods. The aim of this review is to provide a synthesis of research reporting the trauma treatment costs and factors associated with higher treatment costs in high income countries.

**Methods:**

A systematic search for articles relating to the cost of acute trauma care was performed and included studies reporting injury severity scores (ISS), per patient cost/charge estimates; and costing methods. Cost and charge values were indexed to 2011 cost equivalents and converted to US dollars using purchasing power parities.

**Results:**

A total of twenty-seven studies were reviewed. Eighty-one percent of these studies were conducted in high income countries including USA, Australia, Europe and UK. Studies either reported a cost (74.1%) or charge estimate (25.9%) for the acute treatment of trauma. Across studies, the median per patient cost of acute trauma treatment was $22,448 (IQR: $11,819-$33,701). However, there was variability in costing methods used with 18% of studies providing comprehensive cost methods. Sixty-three percent of studies reported cost or charge items incorporated in their cost analysis and 52% reported items excluded in their analysis. In all publications reviewed, predictors of cost included Injury Severity Score (ISS), surgical intervention, hospital and intensive care, length of stay, polytrauma and age.

**Conclusion:**

The acute treatment cost of trauma is higher than other disease groups. Research has been largely conducted in high income countries and variability exists in reporting costing methods as well as the actual costs. Patient populations studied and the cost methods employed are the primary drivers for the treatment costs. Targeted research into the costs of trauma care is required to facilitate informed health service planning.

## Background

The World Health Organisation estimates that approximately 5.8 million people die worldwide each year from injury, accounting for 11% of global mortality [1]. By 2030, road traffic injuries are estimated to be the fifth leading cause of death and the third leading cause of disability worldwide [1]. Injury impacts society significantly on a physical, psychological and economical level [2], costing an estimated US$518 billion globally [3].

Trauma systems have been established in most developed countries [4]. The aim of a trauma system is to facilitate treatment of severely injured patients at a hospital with the appropriate resources. This approach has been demonstrated to significantly reduce trauma patient morbidity in Australia [5-7] and internationally [5,7-9].

In single-payer health systems using episode-funding models, hospitals are reimbursed according to case mix, which may result in underfunding [10-12]. Due to the variability in injury severity, averaging patient costs is problematic and diagnostic-related groups do not adequately represent trauma patient episodes [12]. This is particularly relevant to designated trauma centres, which receive the highest proportion of major trauma patients.

Investigations into economic implications of treating trauma patients have focused on specific injuries, regions and age groups; however there has been no comprehensive synthesis of previous research. Accurate economic data relating to the cost of treating injury and identifying factors of high treatment costs are integral to identifying potential levers for policy makers and planners to design more efficient services. The aim of this study was to provide a synthesis of previous research into the cost of treating trauma from the perspective of the receiving hospital. In doing so, we consider the variation in reported costs according to country, trauma subgroups, predictors of cost and the costing methods employed.

## Methods

### Retrieval of articles

**Figure 1:**
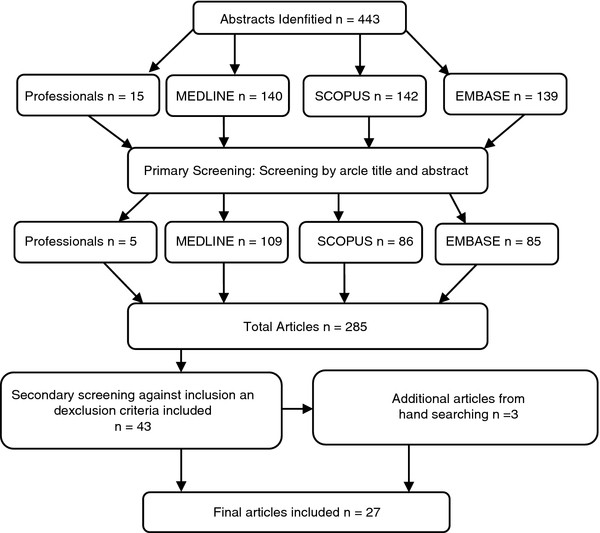
Review flow chart prisma diagram/screening process.

An integrated systematic literature search was conducted to find data that estimated the cost of an acute trauma admission to a designated trauma centre or hospital. The key word search was conducted in Medline, EMBASE, SCOPUS and Google Scholar using adapted search strings (Table 1). Additional searches were conducted in specialised databases such as National Health Service Health Economics Evaluation Database and reports such as the National Health studies and World Health Organisation. Reference lists of identified articles were scanned to extract any additional articles not found using the search string (Figure 1).

**Table 1:** The cost of trauma key word search

**Term**	
**POPULATION**	
#1	exp. Wounds and injuries (MeSH)
#2	((acute or severe) adj3 (trauma or injury or injuries)).mp
**INTERVENTION/EXPOSURE**	
#3	Trauma Centers/
#4	exp. Emergency Service, Hospital/
**OUTCOME**	
#5	Economics, Hospital/
#6	Hospital Costs/
#7	“Costs and Cost Analysis”/
#8	exp Diagnosis-Related Groups/
#9	((cost* or burden*) adj3 (hospital* or “trauma centre*”)).mp
**SEARCH STRATEGY**	
#10	1 OR 2
#11	3 OR 4
#12	5 OR 6 OR 7 OR 8 OR 9
#13	10 AND 11 AND 12
**Total**	**443**

### Article inclusion

Articles were included in the primary screen if they estimated costs or charges of an acute trauma admission in a designated trauma centre or hospital and provided costing methods. Studies were excluded according to the following criteria: 1) were not specific to humans 2) did not relate to an injury/trauma 3) did not capture the acute patient hospitalisation episode in a designated trauma centre OR hospital department 4) did not provide per patient cost or charge estimates for an acute trauma admission and 5) did not provide costing methods and/or analysis.

For the purpose of this review, costs were considered to be based on resource consumption relating to the delivery of health care such as medications, overheads, administration and equipment used by the hospital [13,14]. In contrast, charges refer to a list of prices the hospital allocates for particular services, which includes the costs incurred as well as any losses the hospital makes [13-15].

### Data extraction

Publications that provided per-patient cost or charge data, costing methods and focused on the acute treatment phase of trauma underwent review. Data extracted included: author, year, study aim, study design, location of study, study methods, patient descriptors (age, sex), injury severity score (ISS) [16], type of injury, patient outcomes (length of stay and mortality), cost and charge estimates and cost estimation evaluation, factors associated with cost and reimbursement. No restriction on article language was applied and translations of non-English articles were obtained.

### Secondary article exclusion

Following data extraction by the primary author, a subsequent independent analysis was undertaken by two co-authors (KC & CT). The second screening excluded studies that did not capture the cost of the whole acute episode of hospitalisation (e.g. examined only the intensive care unit (ICU) admission) or provided ISS (which would enable comparison between studies). The ISS is a system of injury stratification based on anatomic diagnosis using the Abbreviated Injury Score [16]. An ISS greater than 15 represents severe injury [16]. Awareness searches were conducted throughout the review to identify further relevant papers. Data were grouped into the subgroups of polytrauma (multiple body regions injured), blunt and penetrating mechanisms.

### Study assessment

The method for estimating cost in each study was assessed, extracted and categorised (Table 2). Costing methods were classified according to which items were included and excluded (e.g. maintenance costs or physician charges), as well as how these items were measured and valued. These items were further grouped into variable and fixed costs. Variable costs were defined as costs that vary with the level of output whereas fixed costs are costs that vary with time rather than quantity [13]. All monetary values were indexed to 2011 cost equivalents of their respective countries. Purchasing power parities adjustments were subsequently used for those studies that did not present original data in US dollars [17-20]. Purchasing power parities is an economic theory used to adjust exchange rates between two countries in order to maintain a constant purchasing power [21].

**Table 2:** The estimation of cost evaluation

**Cost estimation evaluation**	**Number of studies**
**Included**	
**Variable costs**	
Surgical procedures	11
Materials and supply costs	5
Length of stay (A&E, general ward, ICU)	6
Days spent on mechanical ventilation	2
Maintenance	1
Salaries	4
Reimbursements	3
Transportation	2
Laboratory services	8
Radiology services	4
Pharmacy services	2
Emergency services	2
Pathology services	8
**Fixed costs**	
Overheads	4
Depreciation	1
Unspecified	4
**Excluded**	
Physician charges	5
Nursing costs	1
Specialist and ancillary services	3
Non-medical resources	5
Pre-clinical emergency	4
Post patient rehabilitation	3
Food and medication costs	1
General hospital overheads	2
Unspecified	15
**Measured**	
Medical records	9
Trauma registry	12
Audit of medical records	1
Local government departments	1
Health plan database	1
Unspecified	3
**Valued**	
Cost accounting system	7
Reimbursement data	3
Hospital billed charges	2
National cost utilisation project	1
Hospital finance department	7
Local health departments	2
Cost source websites	2
Hospital cost to charge ratio	1
Unspecified	2

### Analysis

Analysis was restricted to descriptive statistics of the costing data. The median, interquartile ranges, means and standard deviation of monetary values were extracted from each study and grouped by mechanism of injury (polytrauma, penetrating and blunt) and injury severity (ISS > 15 and ISS < 15) (Table 3). The data in each subgroup were used to calculate the medians and interquartile ranges.

**Table 3:** Summary of international studies reporting the acute costs of trauma

**Author (year)**	**Country**	**Number of participants (n)**	**Type of trauma**	**Mean ISS**	**Mean cost per trauma admission ($USD)**	**Mean charge per trauma admission ($USD)**
**Thomas (1988)**	USA	340	Polytrauma	26+/-17	33701+/-36843	
**Buckley (1994)**	USA	805	Polytrauma	10.9		16666
**Mock (1994)**	USA	**GSW**: 1116	Penetrating	**GSW**: 13.7		**GSW**:23313+/-42504
		**SW**: 529		**SW**: 7.6		**SW**: 10334+/-117939
**Kizer (1995)**	USA	750	Penetrating	13(1-50)	22115	
				10*	12768*	
**Spaite (1995)**	USA	BAL=>0mg/dL:29	Polytrauma	**Group 1**:10.3		**Group 1**: 11553; (89-209975)
		BAL=0mg/dL:321		**Group 2**: 33		**Group 2**: 1875; (56-71448)
**Goldfarb (1996)**	USA	6963	Polytrauma	18.31+/-6.01		29598
						27349*
**OKeefe (1997)**	USA	12088	Polytrauma Blunt Penetrating	11.2+/-9.1	23399+/-37134	
**Rogers (1997)**	USA	1179	Poiytrauma	96+/-7.8	**ISS 0-16:** 8666	
					**ISS 17-25:** 22979	
					**ISS>25:** 57559	
**Young (1998)**	USA	**Group I (18-64 yr)**: 828	Polytrauma	**Group 1**: 13.8+/-10.7	**Group 1**: 24970 +/-4235	
		**Group 2 (>65yr)**:159		**Group 2**: 162+/-11.1	**Group 2**:21602 +/- 37922	
**Taheri (1998)**	USA	361	Polytrauma	**Group 1**: 14.4+/-0.5	**Group 1**: 15053	
				**Group 2**: 14.7+/-0.5	**Group 2**: 11036	
**Sartorelli (1999)**	USA	1179	Polytrauma	7.2+/-7.6	12988+/-21549	
**Kaya (1999)**	Turkey	347	Polytrauma	13.3+/-0.5		69839
**Taheri (1999)**	USA	696	Polytrauma	>15	14564	
**Rösch (2000)**	Germany	39	Polytrauma	37	111209	
**Dueck (2001)**	Canada	223	Polytrauma	18.8	8196	
					5226*	
**Park (2001)**	USA	204	Polytrauma	**TICU**:25.8+/-7.9	**TICU**: 173013; 119397*	
				**SICU**:27.1+/-9.2	**SICU**:211130; 166653*	
**Schmelz (2002)**	Germany	71	Polytrauma	23	32851	
**Ganzonie (2003)**	Switzerland	35	Polytrauma	33.9 (25-66)	95380	
**Lanzorotti (2003)**	USA	2634	Polytrauma	11.9 +/- 9.2 (1-75)		13096+/- 27003; (837-418553)
**Grotz (2003)**	Germany	103	Polytrauma	29.4	41522	
**Sikand (2005)**	UK	171	Polytrauma	>15	27542; (3216-96558)	
**Small (2006)**	Australia	180	Pedestrian	14.1	22713	
**Davis (2007)**	USA	12615	Blunt Penetrating & TBI	10.67		60094
**Christensen (2008)**	UK	36564	Blunt	**ISS 0-9:** 60%	**ISS 0-9:** 11248; 8471*	
				**ISS 10-16:** 17%	**ISS 10-16:** 16313; 10330	
				**ISS 17-25:** 12%	**ISS 17-25:** 25780; 16123	
				**1SS26-75:** 11%	**ISS 26- 75:** 38426; 27297	
					**Total:** 17295+/-21546; 9782* (6580-17812)	
**Christensen (2008)**	UK	1365	Penetrating	**ISS 1-8:**16%	**ISS 1-8:** 11798;	
				**ISS 9-15:** 50%	8580*	
				**ISS 16-24:** 15%	**ISS 9-15:** 10952; 8568*	
				**ISS 25-45:** 16%	**ISS 16-24:** 17155; 13099*	
				**ISS 46-75:** 41%	**ISS 25-45:** 22408; 13386*	
					**ISS 46-75:** 29832; 19551*	
					**Total:** 14488; 9932* (5976-16034)	
**Zarzaur (2010)**	USA	1914	Blunt	23.3 +/- 7.2	11541 +/-18681 5620*	
**Rowell (2011)**	Australia	72	Polytrauma	28.13+/-9.92	43547 34350*	

*Median costs, Blood alcohol level (*BAL*), Gunshot wounds (*GSW*), Stab wounds (*SW*), Surgical Intensive Care Unit (*SICU*), Trauma Intensive Care Unit (*TICU*).

## Results

The search strategy yielded 443 studies of which 27 were included in the final analysis (Figure 1). Four of these articles were published in non-English languages and were translated into English. Sixteen (61.5%) studies were conducted in USA [22-36], three (11.5%) in the UK [37-39] and Germany [40-42] and two (7.7%) in Australia [43], and one (3.7%) in Switzerland [44] Turkey [45] and Canada [46] respectively (Table 4). Costs and charges were used interchangeably, which created difficulty in evaluating the results of the studies as charges typically vary from costs. Table 4 shows the overall median (interquartile range – IQR) cost of major trauma calculated from the 20 studies (70.1%) reporting cost estimates was $22,448 (IQR $11,819-$33,701). Seven studies (25.9%) reported charges ($26,030; IQR $13,988-$28,199). The median cost of major trauma in the USA was $22,115 (IQR $13,776-$29,335) Australia $33,130 (IQR $27,907-$38,297), UK $18,535 (IQR $11,819 - $25,827) and Germany $41,522 (IQR $37,186 -$76,365).

**Table 4:** Median cost and charge estimates of acute trauma treatment

**Country**	**Number of articles**	**Median $US cost (IQR)**	**Median $US charge (IQR)**
**USA**	16	22,115 (13,776-29,335)	14,881 (8,219- 27,523)
**UK**	3	18,535	
		(11,819-25,827)	
**Germany**	3	41,522 (37,186-76,365)	
**Australia**	2	33,130 (27,907-38,297)	
**Switzerland**	1	95,380	
**Turkey**	1		69,839
**Canada**	1	8,196	
**Total**	**27**	*22,448 (11,819-33,701)	**26,030 (13,988-28,199)

*Median cost (IQR) across studies (all countries).

**Median charge (IQR) across studies (all countries).

### Subgroups associated with costs

**Figure 2:**
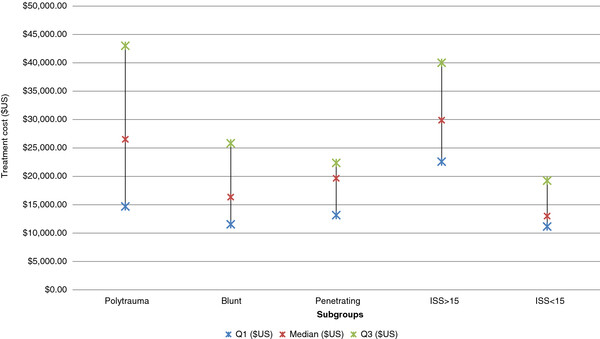
Acute trauma treatment costs per subgroup.

Results varied according to the type of trauma reported. In the polytrauma sub-groups the median cost was $26,521 per patient (IQR $14,686-$43,000). Studies [25,26,37] that reported only penetrating trauma had a median cost of $19,651 per patient (IQR $13,161-$22,365). Two studies [38,47] specifically reported blunt trauma, estimating a median per patient cost of $16,342 (IQR $11,541-$25,827) (Figure 2). Three studies [33,46,48] reported the cost of components associated with treatment costs. These included the cost of staff, emergency department services, pharmaceuticals and radiology.

**Figure 3:**
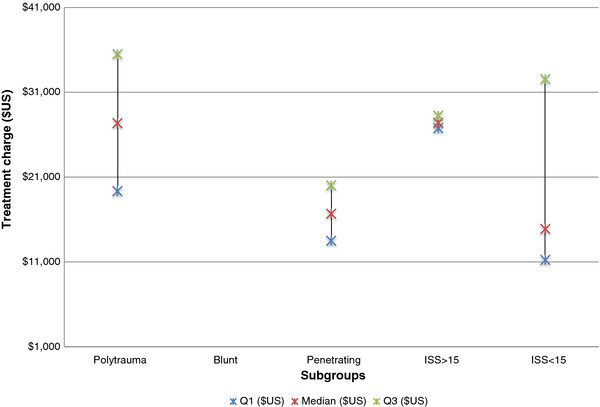
Acute trauma treatment charges per subgroup.

Charge estimates were also reported by type of trauma (Figure 3). The median charge for polytrauma patients was $27,289 (IQR $19,311-$35,417). In patients with penetrating trauma the median charge per patient was $16,666 (IQR $13,500-$19,990).

All studies provided ISS scores to characterise the injury severity of the included patient population. For studies with an average ISS ≥ 15, median cost was $29,886 (IQR $22,581-$40,009) and the median charge was $27,347 (IQR $26,724-$28,199). In studies reporting an average ISS ≤ 15 the median cost was $12,988 (IQR $11,152- $19,229) and median charge was $14,881 (IQR $11,248-$32,508).

### Predictors of cost

Six studies (22%) [22,30,35,37,38,46] used multivariate analyses to determine positive and negative predictors of high treatment costs. Higher treatment costs were associated with polytrauma and specific body regions including the abdomen (OR = 0.65) [35,37,38], chest [46], brain (OR = 0.58) [35]^,^ thorax, spine [10] and upper and lower limbs [38]. Other factors that were found to influence treatment costs included: mode of hospital arrival- the most expensive being helicopter [38], the presence and type of surgical interventions (anaesthesia, neurosurgery and general surgery, wound debridement and intracranial procedures) [22,38,46], the type of treating physician e.g. consultants [38], designated trauma centre level [22], ICU and hospital length of stay [22,30], age [38], road traffic collisions [38] and higher ISS scores [37,38]. Hospital mortality was negatively associated with treatment costs [37,38]. The potential causes of these predictors of high cost were not explained.

### Costing methods

The level of detail provided regarding statistical and costing methods varied between studies. Articles generally provided descriptive analyses of patient cohorts and used a combination of statistical methods. In order to examine differences in cost between groups (such as high and low injury severity), studies used a range of analyses for categorical and continuous outcome measures such as fisher’s exact test and Mann Whitney *U* test. Other studies used simple linear regression analyses [25,29,30,43,45,46] to determine the relationship between cost of care and various patient variables. Studies that further examined the predictors of high treatment costs used multivariate models [37,38] such as multiple logistic regression analysis [34,35,46].

In regards to costing methods, five (18%) studies [27,30,33,39,40] provided comprehensive costing methods which reported details of included and excluded items as well as how these were measured and valued. Seventeen (63%) studies [11,27,30,32-34,37-40,42,43,45,46,48-50] provided information regarding items included in cost or charge estimates and fourteen (52%) studies [22,23,26,27,29,30,33,35,39,40,42,44-46] documented what was excluded from cost or charge estimates. Due to the variation in statistical analysis across studies, a meta-analysis was unable to be performed on the data.

Regarding the source of hospital cost information, twenty-one (78%) studies [23-35,39-42,44,45,47,50] used their respective trauma registry or medical records to derive per patient costs and charges and six (22.2%) studies [22,36-38,43,46] used alternate sources. Alternate sources included local government department estimates [37,38], a health plan database [36] and an audit of medical records [46] (Table 2).

Of the twenty-one (78%) studies using hospital data, nineteen (70%) studies [23-25,27-35,39-42,44,45] used hospital based-accounting systems, of which, 74% reported cost [24,27,29-34,39-42] estimates and 26% reported charges [28,44,45,51]. The five (19%) studies [27,29,31,32,49] that used hospital accounting systems, specified the accounting system methodology. The remaining three studies, [26,47,50] which used hospital data reported costs using cost to charge ratios [47,50] and reimbursement data [26].

## Discussion

This review provides a synthesis of literature concerning the acute treatment costs of trauma in high-income countries and the drivers of higher costs. Results showed the cost of acute treatment was a median of $22,448 across studies (IQR: $11,819-$33,701) and identified factors such as injury severity, surgical interventions, ICU and hospital LOS, were consistently associated with higher treatment costs. Across studies, we identified marked variability in reporting, methods of costing and actual costs and charges [15].

The broad inclusion criteria and time period used in this review resulted in a representative sample, although comparison and ranking of costs between studies was limited due to the variety of costing and statistical methods. The predominance of US data may not be generalisable to universal access health systems such as those in Australia and Canada. Although 90% of the world’s deaths from injuries occur in developing countries [51], there is limited external validity of our results outside the high-income countries, where the reviewed studies were conducted. Further, this is the first review to focus on describing the cost of trauma treatment to hospitals. The economic burden of trauma in post-acute care, requires further investigation [52].

The primary drivers of variations in treatment costs between studies were the variety of patient populations studied and the cost methods employed. Drummond et al. [13] identified two fundamental elements for accurate micro-costing; the measurement of the quantities of resource use and assignment of unit costs or prices. Only five studies provided comprehensive methods behind their cost estimates and the majority of studies collected resource use and unit costs in various ways.

The second driver in the variation in cost estimates between studies identified in our review was the use of cost versus charge estimates. Cost estimates vary in accuracy depending on methods of hospital accounting systems that inform episode funding models, with the consequence that true costs may be under-represented in trauma patients [12,41,53,54]. Conversely, charges generally overestimate actual costs, as hospitals anticipate the trajectory of costs and payments to the hospital each year [55]. As a result, charges do not necessarily give a good indication of what a hospital pays for the resources it consumes in providing services. Adequate funding of trauma centres is essential to enable high quality of care [56] and subsequent patient survival and long term function [57].

Further research focusing on trauma treatment costs and factors associated with higher costs is necessary. Mechanism of injury as a predictor of cost was poorly quantified, some studies reported the high incidents of falls, but did not relate their findings to the cost of treatment. However the association of road trauma with high treatment costs [38] is symptomatic of the global proliferation of road injury [1]. There were inconsistencies associated with high treatment costs and age range, likely due to the variation in ISS and mechanism of injury, although the peak in costs in older patients could be a result of increased falls [58] exacerbated by in-hospital complications and chronic diseases [59]. In comparison to other disease classifications, such as stroke, where the median in-hospital cost is $14,571 (IQR $468 to $146,149) our study demonstrated trauma was substantially more expensive [57].

In an environment of growing cost pressures, new technologies, aging population and consumer expectations, treatment costs and the economic burden to healthcare systems will increase [60]. Accurate economic data is fundamental for improving current funding models and promoting the efficient delivery of services. There remain large gaps in the knowledge around trauma care costs. Health services need to determine if trauma care costs are commensurate with current funding models and funded adequately. Policy makers need to consider if the current modes of trauma care delivery provide sufficient value [60].

## Conclusion

The acute treatment cost of trauma is higher than other disease groups and increases with injury severity, age, surgical intervention and polytrauma. Reported costs differ considerably due to variations in injury type and the inconsistency in study methods. The true costs of trauma to a trauma centre requires further investigation to ensure informed planning and delivery of services.

## Abbreviations

AIS: Abbreviated injury score; ISS: Injury severity score; ICU: Intensive care unit; IQR: Interquartile range.

## Competing interests

CT has a paid part-time position with Novartis Pharmaceuticals.

## Authors’ contributions

LW: performed the literature search analysis, prepared manuscript. KC: literature search, analysis of studies and drafting the manuscript. CT: review design, analysis of studies, reviewed manuscript. SJ: health economist guidance, manuscript preparation. PG: manuscript preparations. JM: manuscript preparations. All authors read and approved the final manuscript.

## Pre-publication history

The pre-publication history for this paper can be accessed here:

http://www.biomedcentral.com/1472-6963/12/267/prepub
